# Prosaposin/Saposin Expression in the Developing Rat Olfactory and Vomeronasal Epithelia

**DOI:** 10.3390/jdb12040029

**Published:** 2024-11-06

**Authors:** Kai Kitamura, Kyoko Saito, Takeshi Homma, Aimi Fuyuki, Sawa Onouchi, Shouichiro Saito

**Affiliations:** 1 Laboratory of Veterinary Anatomy, Joint Graduate School of Veterinary Science, Gifu University, 1-1 Yanagido, Gifu 501-1193, Japan; hataochi@gmail.com (K.K.); shiitakeshi0818@gmail.com (T.H.); fuyuki.aimi.s6@s.gifu-u.ac.jp (A.F.); onouchi.sawa.b6@f.gifu-u.ac.jp (S.O.); 2 Gifu Prefectural Chuo Livestock Hygiene Service Center, 1-1 Yanagido, Gifu 501-1112, Japan; saito-kyoko1@pref.gifu.lg.jp

**Keywords:** immature neuron, olfactory organ, postnatal development, prosaposin, vomeronasal organ

## Abstract

Prosaposin is a glycoprotein widely conserved in vertebrates, and it acts as a precursor for saposins that accelerate hydrolysis in lysosomes or acts as a neurotrophic factor without being processed into saposins. Neurogenesis in the olfactory neuroepithelia, including the olfactory epithelium (OE) and the vomeronasal epithelium (VNE), is known to occur throughout an animal’s life, and mature olfactory neurons (ORNs) and vomeronasal receptor neurons (VRNs) have recently been revealed to express prosaposin in the adult olfactory organ. In this study, the expression of prosaposin in the rat olfactory organ during postnatal development was examined. In the OE, prosaposin immunoreactivity was observed in mature ORNs labeled using olfactory marker protein (OMP) from postnatal day (P) 0. Immature ORNs showed no prosaposin immunoreactivity throughout the examined period. In the VNE, OMP-positive VRNs were mainly observed in the basal region of the VNE on P10 and showed an adult-like distribution from P20. On the other hand, prosaposin immunoreactivity was observed in VRNs from P0, suggesting that not only mature VRNs but also immature VRNs express prosaposin. This study raises the possibility that prosaposin is required for the normal development of the olfactory organ and has different roles in the OE and the VNE.

## 1. Introduction

Prosaposin is a glycoprotein of approximately 60–75 kDa in molecular weight that is widely conserved in vertebrates [1,2,3,4]. Prosaposin is processed into saposins A, B, C, and D in lysosomes [5,6,7,8], and each saposin acts as an essential activator of specific sphingolipid hydrolases in lysosomes [9,10,11,12,13,14,15]. Therefore, prosaposin is a housekeeping protein required for normal lysosomal function [16]. However, prosaposin expression level differs greatly in different types of cell, and thus prosaposin expression level is regulated in tissue- and/or cell-specific manners [17]. Prosaposin is known to be highly expressed in various neurons in the central nervous system [18] and peripheral nervous system [19], and prosaposin may play a role in active autophagy in these neurons as it has been shown that prosaposin deficiency decreases autophagy flux [20,21]. Autophagy is known to elicit cytoprotective effects via the removal of protein aggregates and harmful organelles [22] and thus plays crucial roles in the maintenance of postmitotic neurons [23,24,25]. Therefore, prosaposin appears to play a crucial role in various kinds of mature neurons by providing saposins. Furthermore, prosaposin that has not been processed into saposins exists in various body fluids including seminal plasma, bile, pancreatic juice, milk, and cerebrospinal fluid and serum [6,26,27,28]. Prosaposin is also thought to be secreted in an autocrine/paracrine manner in some neurons [29,30]. Extracellular prosaposin is known to act as a neurotrophic factor [31] via G protein-coupled receptors [32,33,34,35]. In summary, prosaposin can affect the homeostasis of neurons from inside and/or outside the cells.

In many mammals, including rodents, the olfactory organ is composed of the olfactory epithelium (OE) and the vomeronasal epithelium (VNE) [36,37]. The OE is a pseudostratified epithelium composed of supporting cells (SCs), olfactory receptor neurons (ORNs), and basal cells (BCs) [38,39,40]. The VNE is a neuroepithelium covering the medial wall of the vomeronasal organ (VNO) and is composed of SCs and vomeronasal receptor neurons (VRNs) [41,42,43]. In the adult VNO, BCs are mainly located in the marginal region of the VNE, which is the boundary between the neuronal epithelium and non-neuronal epithelium, and supply immature VRNs [44,45,46,47]. The OE and the VNE are known to show active neurogenesis, not only during development but also in the adult stage [44,47,48,49]. The expression pattern of prosaposin in the vertebrate olfactory system remains unknown, and we previously reported that there was a high expression level of prosaposin in mature receptor neurons, but not in immature neurons or progenitor BCs, in the adult mouse olfactory organ [50]. That study suggested that prosaposin has a function in mature receptor neurons. On the other hand, it was reported by Fujita et al. [51] and Oya et al. [52] that about half of prosaposin knockout mice died in utero or within the first 1–2 days after birth and that the remaining mice grew normally until 18–20 days after birth, but then began to show neurological symptoms and did not survive beyond 40 days after birth. Those studies suggested that prosaposin has a significant role in the normal development of neurons. To consider the role of prosaposin in the development of the olfactory organ, the expression of prosaposin compared to the distribution pattern of mature neurons that express olfactory marker protein (OMP) in the rat olfactory organ during the postnatal period was examined in this study.

## 2. Materials and Methods

### 2.1. Animals and Tissue Preparation

The experimental procedures in this study were reviewed by the Committee for Animal Research and Welfare of Gifu University and received final approval from the President of the University (Permission No. 2020-257, 02/04/2021). The Gifu University regulations conform to the Japanese Act on Welfare and Management of Animals, and Standards Relating to the Care and Keeping and Reducing Pain of Laboratory Animals (Notice of the Ministry of the Environment No. 88, 2006).

Wistar rats of both sexes were sacrificed on postnatal day 0 (P0), P10, P20, P30, P40, and P60. Three rats of both sexes were used on each postnatal day. After euthanasia with an intraperitoneal injection of sodium pentobarbital (50 mg/kg), the rats were perfused transcardially with physiological saline, followed by 4% paraformaldehyde, in 0.1 M phosphate-buffered saline (PBS; pH 7.4). The OE with the ethmoid bone, the nasal concha, and the nasal septum and the VNO with the vomeronasal cartilage were dissected out, fixed in the same fixative solution at 4 °C overnight, decalcified by immersion in 200 mM ethylenediamine tetraacetic acid (EDTA) in 0.1 M phosphate buffer (pH 7.4) for 1 week, routinely embedded in paraffin, cut coronally at 5 µm in thickness, and mounted onto poly L-lysine and MAS-coated slides (Matsunami Glass Ind., Ltd., Osaka, Japan).

### 2.2. Immunohistochemistry

Immunohistochemistry was performed for prosaposin as reported previously [19,53]. Briefly, after deparaffinization and rinsing in 0.01M PBS, the sections were incubated with 2% normal goat serum (Sigma-Aldrich, St. Louis, MO, USA) for 30 min. After being rinsed again in PBS, the sections were incubated with a rabbit anti-saposin C domain antibody (Table 1), generated previously [18,54] and diluted 1:250, at 4 °C overnight. After rinsing in PBS, the sections were incubated with biotinylated goat anti-rabbit IgG (Dako, Glostrup, Denmark) at a 1:500 dilution with immunostaining buffer for 30 min. The sections were again rinsed in PBS and incubated with VECTASTAIN ABC reagent (Vector, Burlingame, CA, USA) for 30 min. Finally, the sections were colorized with 500 mM Tris-HCl (pH 7.6) containing 0.01% 3,3′-diaminobenzidine tetrahydrochloride and 0.003% H_2_O_2_ for 10 min, rinsed under running water and in distilled water, dehydrated using a graded ethanol series and xylene, and coverslipped. For OMP immunostaining, the sections were incubated with 2% normal rabbit serum followed by incubation overnight at 4 °C with goat anti-OMP antibody (Table 1; 019-22291; Fujifilm Wako Chemicals, Osaka, Japan) diluted 1:200, and then biotinylated rabbit anti-goat IgG (Abcam, Cambridge, UK) diluted 1:500 was applied and the sections were colorized as described above. Negative controls were created by replacing the primary antibody with normal rabbit IgG (Fujifilm Wako Pure Chemical Co.), and no specific staining was observed in the control sections (Supplemental Figure S1).

### 2.3. Immunofluorescence

Immunofluorescence was performed, as reported previously [50], with some modification. For example, the antibodies for prosaposin/saposin used in this study were different from those used in a previous report [50], and the antibody used in this study did not require the antigen retrieval treatment. Briefly, the sections were incubated with 1% blocking reagent (Roche) and 2% normal donkey serum at room temperature (RT) for 30 min, followed by incubation with a mixture of rabbit anti-saposin C domain antibody (Table 1), diluted 1:250, and goat anti-OMP antibody (Table 1), diluted 1:200, at 4 °C overnight. After rinsing with PBS, a mixture of Alexa Fluor 594-conjugated donkey anti-rabbit IgG, diluted 1:500, and Alexa Fluor 488-conjugated donkey anti-goat IgG, diluted 1:500, was applied to the sections at RT for 60 min. Then, the sections were counterstained with DAPI (Dojindo Laboratories, Kumamoto, Japan), diluted 1:1000, at RT for 10 min; cover-slipped; and observed under a fluorescence microscope (Z-X810; Keyence, Osaka, Japan). Negative controls were created by replacing the primary antibody with normal rabbit IgG (Fujifilm Wako Pure Chemical Co.) and no specific staining was observed in the control sections.

## 3. Results

### 3.1. Prosaposin Immunoreactivity in the OE

The anti-saposin C domain antibody used in this study can detect both saposin C and its precursor prosaposin, but its immunoreactivity was referred to as “prosaposin immunoreactivity” for convenience. We confirmed that the expression pattern of prosaposin mRNA in the adult rat olfactory organ was the same as that in the adult mouse olfactory organ analyzed previously [50] by in situ hybridization, and prosaposin expression in the adult rat olfactory organ was also confirmed by Western blot analysis (Supplemental Figure S2).

**Figure 1:**
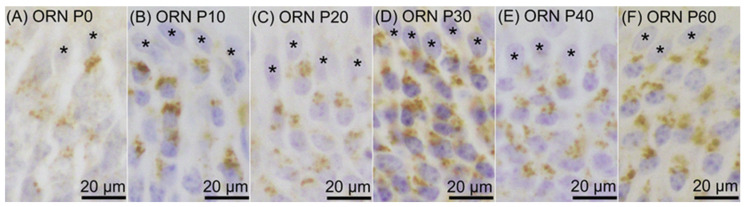
Prosaposin immunoreactivity in olfactory receptor neurons (ORNs) on postnatal day (P) 0 (**A**), P10 (**B**), P20 (**C**), P30 (**D**), P40 (**E**), and P60 (**F**). Asterisks indicate supporting cells showing no immunoreactivity. The sections were counterstained with hematoxylin. Bars are 20 μm.

**Figure 2:**
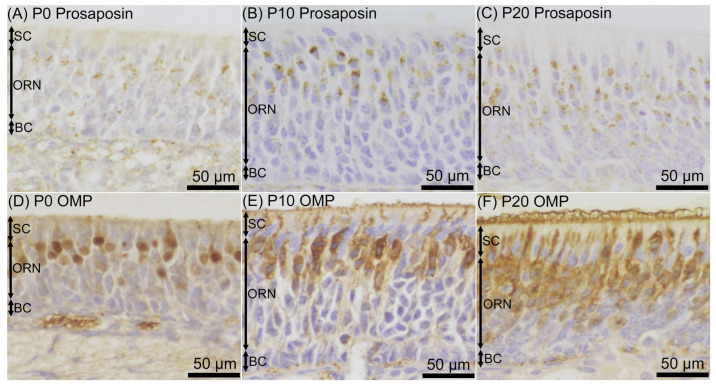
Prosaposin (**A**–**C**) and olfactory marker protein (OMP) (**D**–**F**) immunoreactivity in the olfactory epithelium on postnatal day (P) 0 (**A**,**D**), P10 (**B**,**E**), and P20 (**C**,**F**). The distribution of prosaposin-positive olfactory receptor neurons (ORNs) was similar to that of OMP-positive ORNs at each time point. The sections were counterstained with hematoxylin. Bars are 50 μm. BC, basal cell; SC, supporting cell.

**Figure 3:**
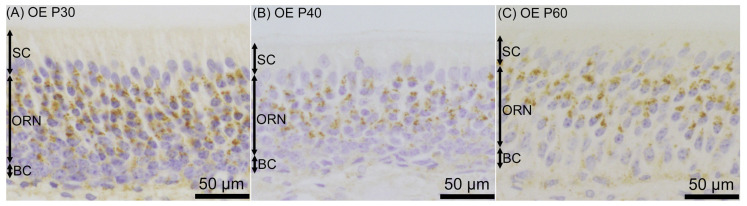
Prosaposin immunoreactivity in the olfactory epithelium (OE) in the late postnatal period, on postnatal day (P) 30 (**A**), P40 (**B**), and P60 (**C**). The distribution of prosaposin-positive olfactory receptor neurons (ORNs) was similar to that on P20 at each examined time point. Bars are 50 μm. BC, basal cell; SC, supporting cell.

**Figure 4:**
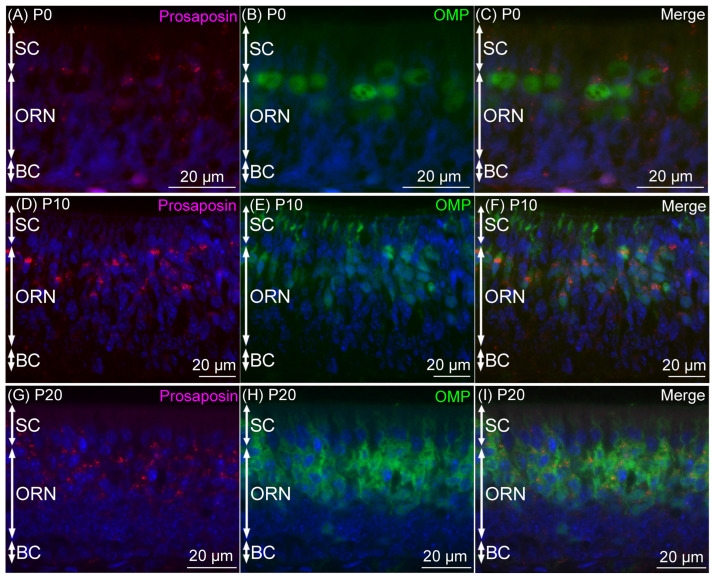
Double immunofluorescence results of prosaposin (red) and olfactory marker protein (OMP) (green) counterstained with DAPI (blue) in the olfactory epithelium on postnatal day (P) 0 (**A**–**C**), P10 (**D**–**F**), and P20 (**G**–**I**). Prosaposin immunoreactivity was observed in OMP-positive olfactory receptor neurons (ORNs) at each examined time point. Bars are 20 μm. BC, basal cell; SC, supporting cell.

Prosaposin immunoreactivities were observed as dot-like structures in cell bodies of a certain number of ORNs throughout the examined period, from P0 to P60 (Figure 1). On P0, prosaposin-positive ORNs with moderate immunointensity were located in the apical half region of the ORN layer (Figure 2A), and the distribution pattern of prosaposin-positive ORNs was similar to that of OMP-positive ORNs (Figure 2D). The distribution pattern of prosaposin-positive ORNs on P10 was similar to that on P0, with more intense immunoreactivity (Figure 2B), and was also similar to the distribution pattern of OMP-positive ORNs (Figure 2E). On P20, prosaposin-positive ORNs were located in the apical 2/3 of the ORN layer (Figure 2C), and this distribution pattern was similar to that of OMP-positive ORNs (Figure 2F). The distribution pattern of prosaposin immunoreactivity in the ORN layer was almost the same from P20 to P60 (Figure 3). Immunofluorescence was performed to examine the similar distribution patterns of prosaposin and OMP immunoreactivities (Figure 4). Prosaposin immunoreactivities were observed in OMP-positive cells on P0, P10, and P20. Prosaposin immunoreactivity was not observed in SCs or BCs throughout the examined period, and the reginal differences in prosaposin immunoreactivity could not be observed in the OE.

### 3.2. Prosaposin Immunoreactivity in the VNE

**Figure 5:**
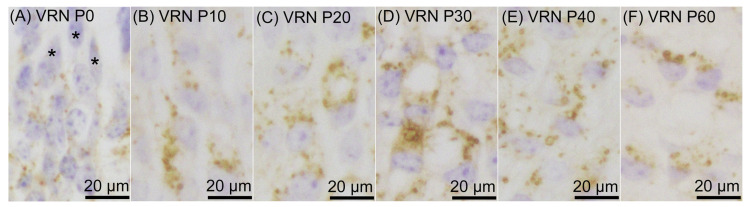
Prosaposin immunoreactivity in vomeronasal receptor neurons (VRNs) on postnatal day (P) 0 (**A**), P10 (**B**), P20 (**C**), P30 (**D**), P40 (**E**), and P60 (**F**). Asterisks indicate supporting cells showing no immunoreactivity. The sections were counterstained with hematoxylin. Bars are 20 μm.

**Figure 6:**
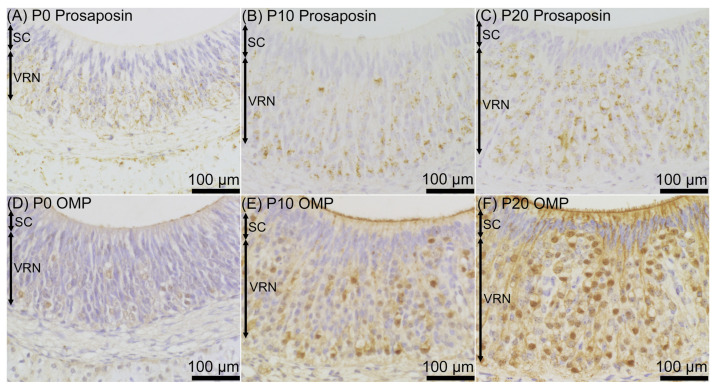
Prosaposin (**A**–**C**) and olfactory marker protein (OMP) (**D**–**F**) immunoreactivity in the vomeronasal epithelium on postnatal day (P) 0 (**A**,**D**), P10 (**B**,**E**), and P20 (**C**,**F**). On P0, weak prosaposin immunoreactivity was observed throughout the layer of vomeronasal receptor neurons (VRNs) (**A**), but OMP immunoreactivity was hardly observed (**B**). On P10, in addition to weak immunoreactivity throughout the VRN layer, intense prosaposin immunoreactivity was observed in VRNs located in the basal region of the VRN layer (**B**), and the distribution of these neurons was similar to that of OMP-positive VRNs (**E**). On P20, intense prosaposin immunoreactivity (**C**) as well as OMP immunoreactivity (**F**) were observed throughout the VRN layer. Bars are 100 μm. SC, supporting cell.

**Figure 7:**
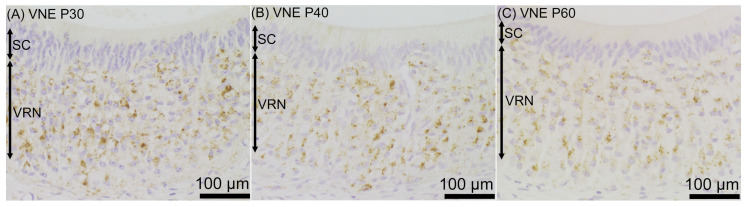
Prosaposin immunoreactivity in the vomeronasal epithelium (VNE) in the late postnatal period, on postnatal day (P) 30 (**A**), P40 (**B**), and P60 (**C**). The distribution of prosaposin-positive vomeronasal receptor neurons (VRNs) was similar to that on P20 at each examined time point. Bars are 100 μm. SC, supporting cell.

**Figure 8:**
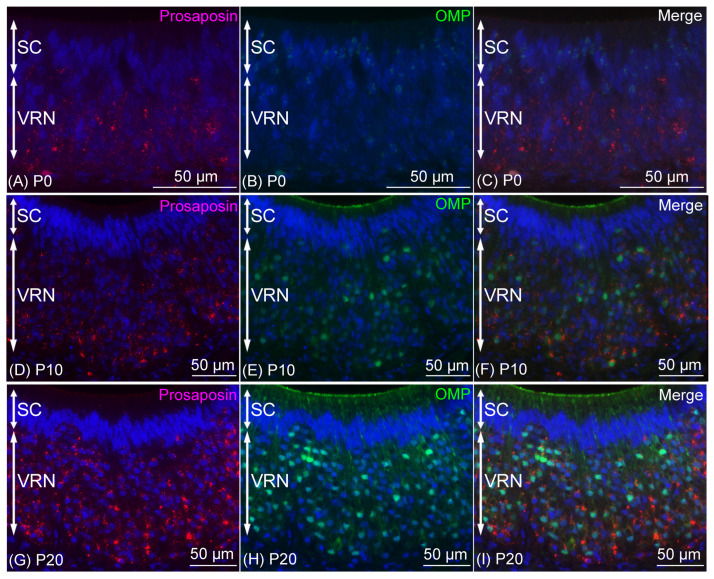
Double immunofluorescence results of prosaposin (red) and olfactory marker protein (OMP) (green) after being counterstained with DAPI (blue) in the vomeronasal epithelium on postnatal day (P) 0 (**A**–**C**), P10 (**D**–**F**), and P20 (**G**–**I**). On P0, prosaposin immunoreactivity, but not OMP immunoreactivity, was observed in the layer of vomeronasal receptor neurons (VRNs) (**A**–**C**). On P10, intense prosaposin immunoreactivity was observed in OMP-positive VRNs (**D**–**F**). On P20, intense prosaposin immunoreactivity as well as OMP immunoreactivity were observed throughout the VRN layer (**G**–**I**). Bars are 50 μm. SC, supporting cell.

Prosaposin immunoreactivities were observed as dot-like structures in the cell bodies of VRNs throughout the examined period, from P0 to P60 (Figure 5). On P0, prosaposin immunoreactivities with weak immunointensity were observed in almost all of the VRNs in the VNE (Figure 6A), while OMP immunoreactivity was not found in the VNE (Figure 6D). On P10, weak prosaposin immunoreactivity was observed throughout the VRN layer, except for the notable intense prosaposin immunoreactivity in a small number of VRNs. These prosaposin-positive VRNs were primarily located in the basal region of the VRN layer (Figure 6B). The distribution pattern of intensely prosaposin-positive VRNs was similar to that of OMP-positive VRNs (Figure 6E). On P20, intense immunoreactivities of prosaposin were observed in VRNs throughout the VRN layer (Figure 6C), and the distribution pattern of prosaposin-positive VRNs was similar to that of OMP-positive VRNs (Figure 6F). From P20 to P60, the distribution pattern of immunoreactivity was almost the same (Figure 7). Prosaposin immunointensity seemed to increase during postnatal development, but further experiments are required to confirm this. When performing double immunofluorescence for prosaposin and OMP, on P0, only prosaposin immunoreactivity was observed in the VRN layer (Figure 8A–C). On P10, prosaposin immunoreactivities were obvious in OMP-positive VRNs located in the basal region of the VRN layer (Figure 8D–F). On P20, prosaposin immunoreactivities were observed in OMP-positive VRNs distributed widely in the VRN layer (Figure 8G–I). Prosaposin immunoreactivity was not observed in SCs throughout the examined period.

There was no sex difference in the immunostaining pattern, and immunoreactivity was not observed in the section in which normal rabbit serum was applied instead of the primary antibody on each of the postnatal days in both the OE and the VNE (Supplemental Figure S1).

## 4. Discussion

This study is the first to reveal the expression of prosaposin in developing rat olfactory organs. It was shown that mature ORNs and VRNs have high expression levels of prosaposin, and that immature VRNs also express prosaposin.

In the rat OE examined in this study, the distribution pattern of prosaposin-immunoreactive ORNs during postnatal development was similar to that of OMP-positive neurons. Prosaposin-positive ORNs were distributed in the apical half region of the ORN layer on P0 and P10, and an adult-like distribution pattern of prosaposin-positive ORNs was observed from P20, matching the distribution pattern of OMP-positive neurons in the rat developing OE, as demonstrated in this study and in other studies [56,57,58]. OMP immunoreactivity was reported to be observed in the nucleoplasm in the earlier postnatal stages [56] and a similar staining pattern was also observed on P0 in this study. The expression ratio of prosaposin/saposin in ORNs is uncertain because the antibody used in this study can detect both prosaposin and saposins [54], but the results obtained in this study may indicate that the expression of prosaposin/saposins is one of the fundamental features of mature ORNs. Mature ORNs require a larger amount of prosaposin/saposins, needing more than is required by immature ORNs, SCs, and BCs. This applies not only in the adult stage but also during postnatal development. It is therefore thought that the expression of prosaposin/saposins is required to maintain the homeostasis of mature ORNs, rather than to stimulate cell growth and/or proliferation in the OE.

In the OE, prosaposin immunoreactivity was mainly observed in the supranuclear region of ORNs, where there is the greatest volume of cytoplasm containing the endoplasmic reticulum, Golgi apparatus, numerous vesicles, endosomes, and lysosomes [59]. It was uncertain in this study whether prosaposin exists in vesicles, endosomes, and/or lysosomes and thus performs functions, or whether it is kept in the endoplasmic reticulum and/or Golgi apparatus and thus is in a state of preparing to function in OMP-positive ORNs at the early postnatal stage. This should be examined by electron microscopic observation to determine the functional significance of prosaposin during postnatal development.

In the rat VNE examined in this study, prosaposin-immunoreactive VRNs were also observed throughout the examined postnatal period. On P0, prosaposin-positive VRNs were observed throughout the VRN layer, but OMP-positive VRNs were hardly observed, as was reported in another study [56]. The rat VNO has been reported to show morphological maturation around P21 [60], and progenitor cells and immature VRNs are thus widely distributed throughout the VNE on P0 [61]. OMP-positive neurons have been reported to appear after P4 in the VNE, in contrast to the results on embryonic day 14 in the OE [56]. Prosaposin immunoreactivity on P0 may indicate that immature VRNs express prosaposin weakly but more abundantly than immature ORNs. In adults, neuronal proliferation in the VNE is known to be slow compared to that in the OE [61,62]. It takes a long time for immature VRNs generated in the marginal region of the VNO to migrate to the appropriate position [44,47]. Therefore, prosaposin expression in immature VRNs on P0 may indicate that they have longer survival and greater migration than immature ORNs. In contrast to immature ORNs, it is difficult to detect immature VRNs in the normal adult VNO by light microscopy because they are distributed sparsely. Therefore, in our previous study [50], we could not determine the expression level of prosaposin in immature VRNs, and this developmental study suggests that there are different expression levels of prosaposin in immature neurons in the OE and the VNE.

On P10, in addition to weak prosaposin immunoreactivity throughout the VNE, some VRNs located in the basal region of the VNE showed intense prosaposin immunoreactivity in this study. A few VRNs located in the middle of the apical region of the VNE also showed intense immunoreactivity, but these VRNs are also thought to be neurons situated near the basement membrane of the VNE. In rodents, capillaries intrude in the VNE, and the basement membrane is thus highly undulated in the VNE [61,63]. The distribution of these prosaposin-positive VRNs showing intense immunoreactivity matched that of OMP-positive VRNs in this study. VRNs located in the basal region of the VNE appear to mature before the maturation of VRNs in the apical region of the VNE because G protein α subunit Gαo (expressed specifically in basally located mature VRNs) begins to be expressed earlier than Gαi2 (expressed specifically in apically located mature VRNs) in the VNE during postnatal development [64]. These results suggest that prosaposin/saposins play a pivotal role in mature VRNs, similar to its function in mature ORNs. From P20, in this study, it was shown that intense prosaposin immunoreactivity was distributed widely in the VRN layer and that OMP-positive VRNs also occupied the VRN layer. These results also match the results for the adult mouse VNE, showing that OMP-positive VRNs express prosaposin intensely [50]. Together with the fact that the accessory olfactory bulb which receives axons from the VNE reaches electrophysiological maturity at P18 in rats [65], the adult-like expression profile of prosaposin may be established around P20 in the rat VNO.

This study suggests that prosaposin plays an important role in the development and maintenance of the olfactory organ. Together with the fact that the administration of prosaposin can reduce the neurodegeneration that occurs in peripheral nerve dysfunction [66], ischemia [67,68,69], Parkinson’s disease [70], and Alzheimer’s disease [71], this study also suggests that prosaposin administration may be effective for the treatment of olfactory neurodegeneration.

## 5. Conclusions

Prosaposin was revealed to be expressed in mature ORNs and VRNs during postnatal development, suggesting that prosaposin is required for the maintenance of the olfactory organ after birth. It was also revealed that immature VRNs express prosaposin, suggesting a relationship between prosaposin and the unique dynamics of immature VRNs. The influence of prosaposin in the development and maintenance of neurons may differ between the OE and the VNE, but prosaposin may function as one of the pivotal molecules in both epithelia and thus it is expected that prosaposin administration will be an effective treatment for olfactory organ disorders.

## Figures and Tables

**Table 1:** Primary antibodies used in this study.

Name	Specificity	Antigen	Manufacturer, ID, Host	Dilution	Reference
Anti-saposin C domain antibody	Both prosaposin and saposin C	Saposin C extracted from bovine spleen	Generated by Sano et al. (1989), Rabbit, [54]	1:250	Sano et al. (1989) Biochem. Biophys. Res. Commun. 165, 1191–1197. [54] Kondoh et al. (1993) J. Comp. Neurol. 334, 590–602. [18]
Anti-OMP antibody	Olfactory marker protein (OMP)	Rodent OMP	Fujifilm Wako Pure Chemical Co. (Osaka, Japan), 019-22291, Goat	1:200	Koo et al. (2004) J. Neurochem. 90, 102–116. [55]

## Data Availability

Data will be made available on request.

## Supplementary Materials

The following supporting information can be downloaded at: https://www.mdpi.com/article/10.3390/jdb12040029/s1, Figure S1: Negative controls for the immunohistochemistry; Figure S2: Prosaposin expression examined by in situ hybridization and Western blot in the adult rat olfactory and vomeronasal epithelia.

## Abbreviations

BC	basal cell
OE	olfactory epithelium
OMP	olfactory marker protein
ORN	olfactory receptor neuron
P	postnatal day
SC	supporting cell
VNE	vomeronasal epithelium
VNO	vomeronasal organ
VRN	vomeronasal receptor neuron
