# Supplementary material for: Prosaposin/Saposin Expression in the Developing Rat Olfactory and Vomeronasal Epithelia
Source: J Dev Biol. 2024 Nov 6;12(4):29. doi: 10.3390/jdb12040029 (PMC11587001; doi:10.3390/jdb12040029)
Supplement: Supplementary file 1 [file jdb-12-00029-s001.zip › jdb-3208413-supplementary/Figure S1.pdf]

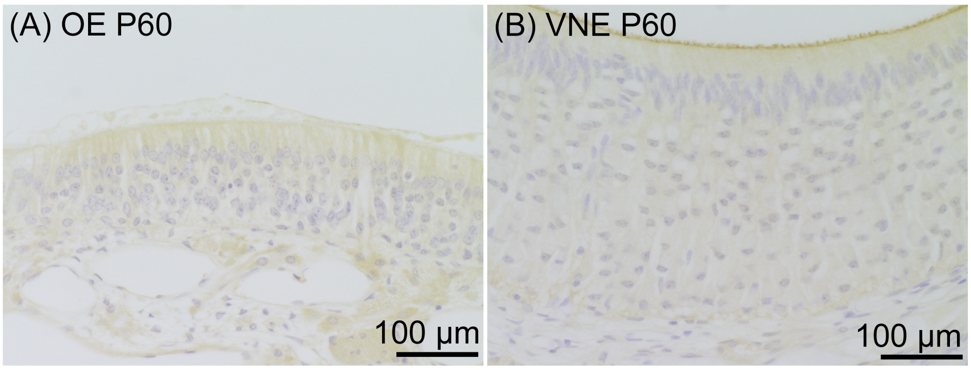

Supplemental figure 1. Immunoreactivity using normal rabbit IgG instead of the primary antibody in (A) the olfactory epithelium (OE) and (B) the vomeronasal epithelium (VNE), on postnatal day (P) 60.
