# Supplementary material for: Prosaposin/Saposin Expression in the Developing Rat Olfactory and Vomeronasal Epithelia
Source: J Dev Biol. 2024 Nov 6;12(4):29. doi: 10.3390/jdb12040029 (PMC11587001; doi:10.3390/jdb12040029)
Supplement: Supplementary file 1 [file jdb-12-00029-s001.zip › jdb-3208413-supplementary/Figure S2.pdf]

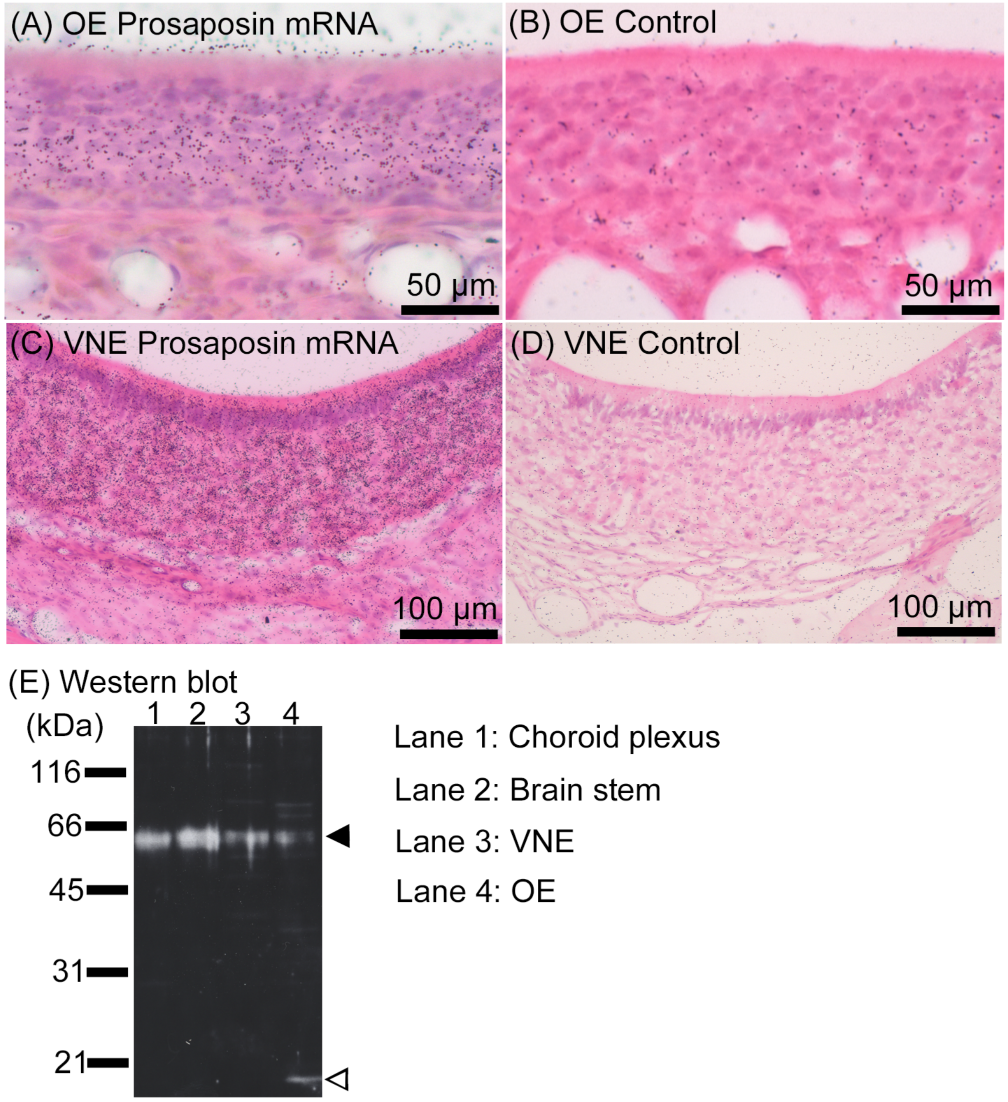

Supplemental figure 2. *In situ* hybridization for prosaposin mRNA using radioisotope-labeled antisense (A and C) and sense (B and D) probes in the adult rat olfactory epithelium (OE) (A and B) and vomeronasal epithelium (VNE) (C and D), with hematoxylin and eosin staining. Prosaposin mRNA expression was observed in mature olfactory receptor neurons in the OE, and vomeronasal receptor neurons and supporting cells in the VNE. This distribution pattern was same to that in adult mouse OE and VNE we reported previously [50]. For the methodology and the probe information, please see our previous report [53]. (E) Western blot of adult rat organs stained with prosaposin antibody used in this study. The antibody showed the band at the position of approximately 66 kDa (black arrowhead), which is the estimated molecular weight of prosaposin, in the choroid plexus, brain stem, VNE and OE. In the OE, an additional band at the position of less than 21 kDa (white arrowhead), which is thought to be saposin, was also observed For the methodology, please see our previous report [53].
